# Supplementary material for: Structure, gating, and pharmacology of human CaV3.3 channel
Source: Nat Commun. 2022 Apr 19;13:2084. doi: 10.1038/s41467-022-29728-0 (PMC9019099; doi:10.1038/s41467-022-29728-0)
Supplement: Supplementary file 1 — Supplementary Information [file 41467_2022_29728_MOESM1_ESM.pdf]

Supplementary Information

Structure, gating and pharmacology of human $Ca_V3.3$ channel

Lingli He[1,2,3]*, Zhuoya Yu[1,2,3]*, Ze Geng[4,5]*, Zhuo Huang[4,5]*, Changjiang Zhang[2,3], Yanli Dong[1,2], Qihao Chen[1,2,3], Le Sun[6], Xinyue Ma[4,5], Bo Huang[7], Xiaoqun Wang[2,3], Yan Zhao[1,2,3]**

[1] National Laboratory of Biomacromolecules, CAS Center for Excellence in Biomacromolecules, Institute of Biophysics, Chinese Academy of Sciences, Beijing 100101, China

[2] State Key Laboratory of Brain and Cognitive Science, Institute of Biophysics, Chinese Academy of Sciences, 15 Datun Road, Beijing, 100101, China

[3] College of Life Sciences, University of Chinese Academy of Sciences, Beijing 100049, China

[4] State Key Laboratory of Natural and Biomimetic Drugs, Department of Molecular and Cellular Pharmacology, School of Pharmaceutical Sciences, Peking University Health Science Center, Beijing, 100191, China

[5] IDG/McGovern Institute for Brain Research, Peking University, Beijing, 100871, China

[6] Beijing Institute of Brain Disorders, Capital Medical University, Beijing, 100069, China

[7] StoneWise Ltd., 1708, Block B, No.19 Zhongguancun Street, Haidian District, Beijing, China

* These authors contribute equally to this project.

** Correspondence emails: zhaoy@ibp.ac.cn (Y.Z.)

Supplementary Figures

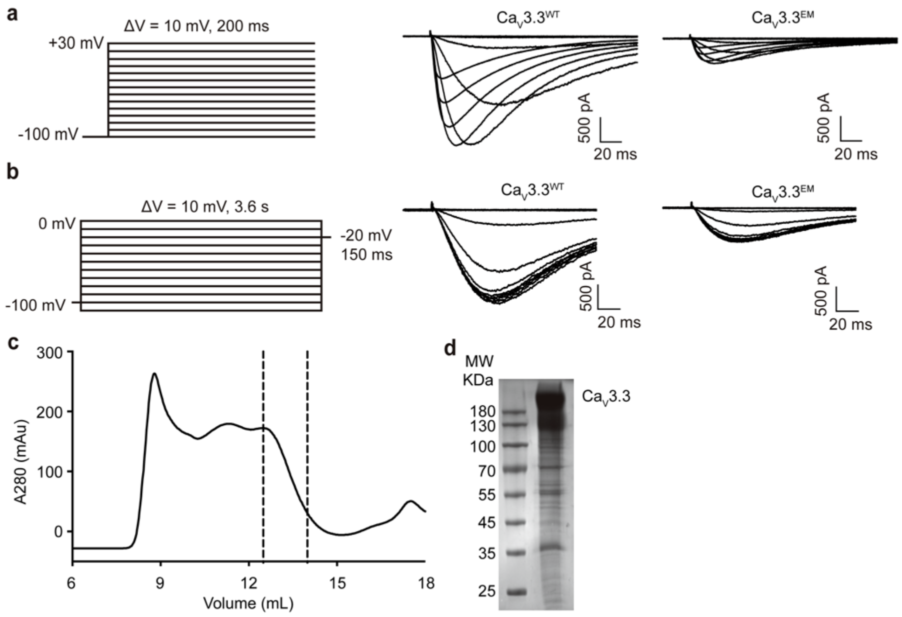

Supplementary Fig. 1 Functional characterization and purification of the Ca$_V$3.3.

a. Standard voltage protocol used for activation and representative whole-cell voltage-clamp Ca$_V$3.3^{WT} and Ca$_V$3.3^{EM} current traces obtained from a series of 200ms voltage steps from –100 mV to +30 mV in 10 mV increments. **b.** Standard voltage protocol used for inactivation and typical whole-cell voltage-clamp Ca$_V$3.3^{WT} and Ca$_V$3.3^{EM} current traces assessed with a 3.6s holding-voltages ranging from −110 mV to 0 mV (10 mV increments) followed by a 150ms test pulse at -20 mV. **c**. Representative elution profile of the purified Ca$_V$3.3 proteins by size-exclusion chromatogram (Superose 6 increase). Peak fractions (marked within black dashed lines) are pooled and concentrated for cryo-EM study. **d**. Corresponding Coomassie blue-stained SDS-PAGE gel for Ca$_V$3.3 proteins used in cryo-EM study. The experiments were repeated independently with more than 3 times with similar results.

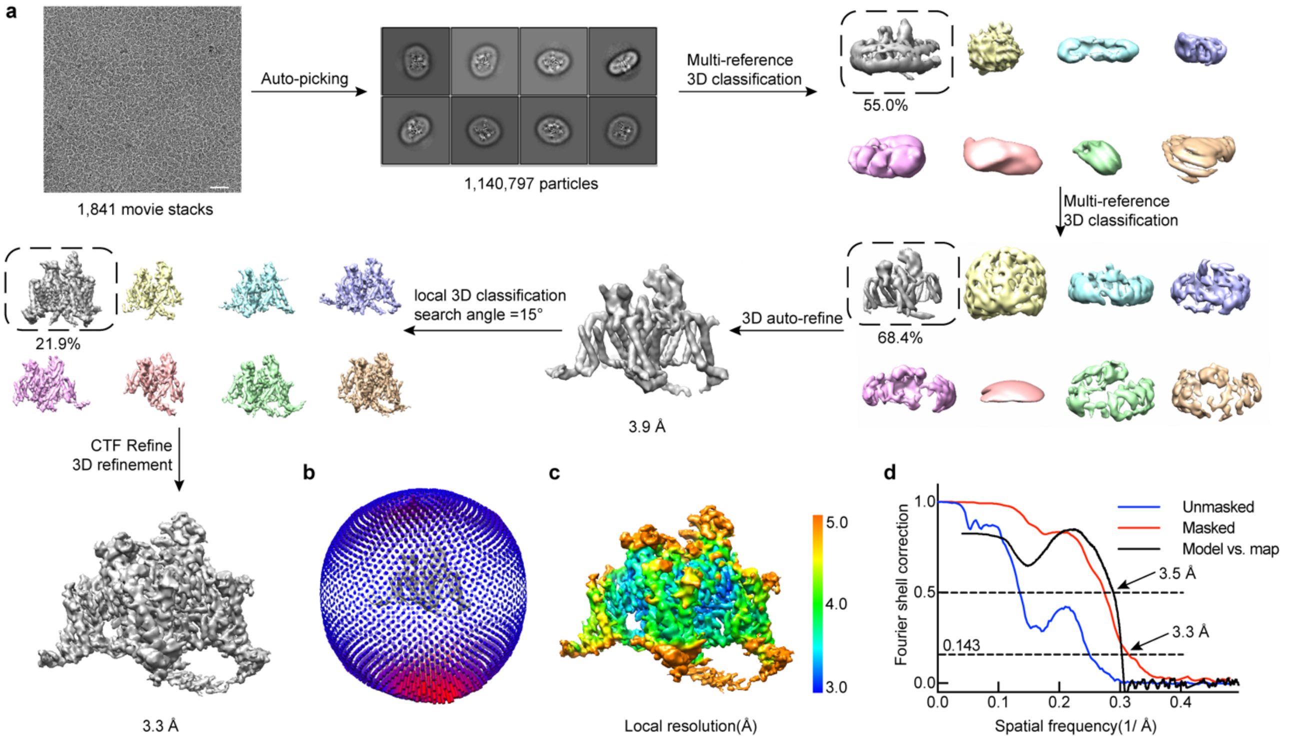

Supplementary Fig. 2 Cryo-EM data processing of $Ca_V3.3^{apo}$

a. Flowchart of cryo-EM data processing. Representative raw cryo-EM micrograph of a total of 1841 movie stacks and 2D class averages shown distinct secondary structure features from different views of $Ca_V3.3^{apo}$, respectively. Several rounds of 3D classifications are conducted to clean particles, followed by Bayesian Polish and CTF Refine to improve image quality. Details can be found in Materials and Methods. The final map was reported at 3.3 Å according to the GSFSC criterion. **b.** The angular distribution of the final reconstruction. The height of each spike indicated the number of particles in the designated orientation. **c.** Electron density map colored by local resolution values. **d.** Fourier Shell Correlations (FSC) of the final map of the $Ca_V3.3^{apo}$ complex, calculated between two independently refined half-maps before (blue) and after (red) post-processing. The FSC curve calculated between the cryo-EM density map and the structural model are shown in black.

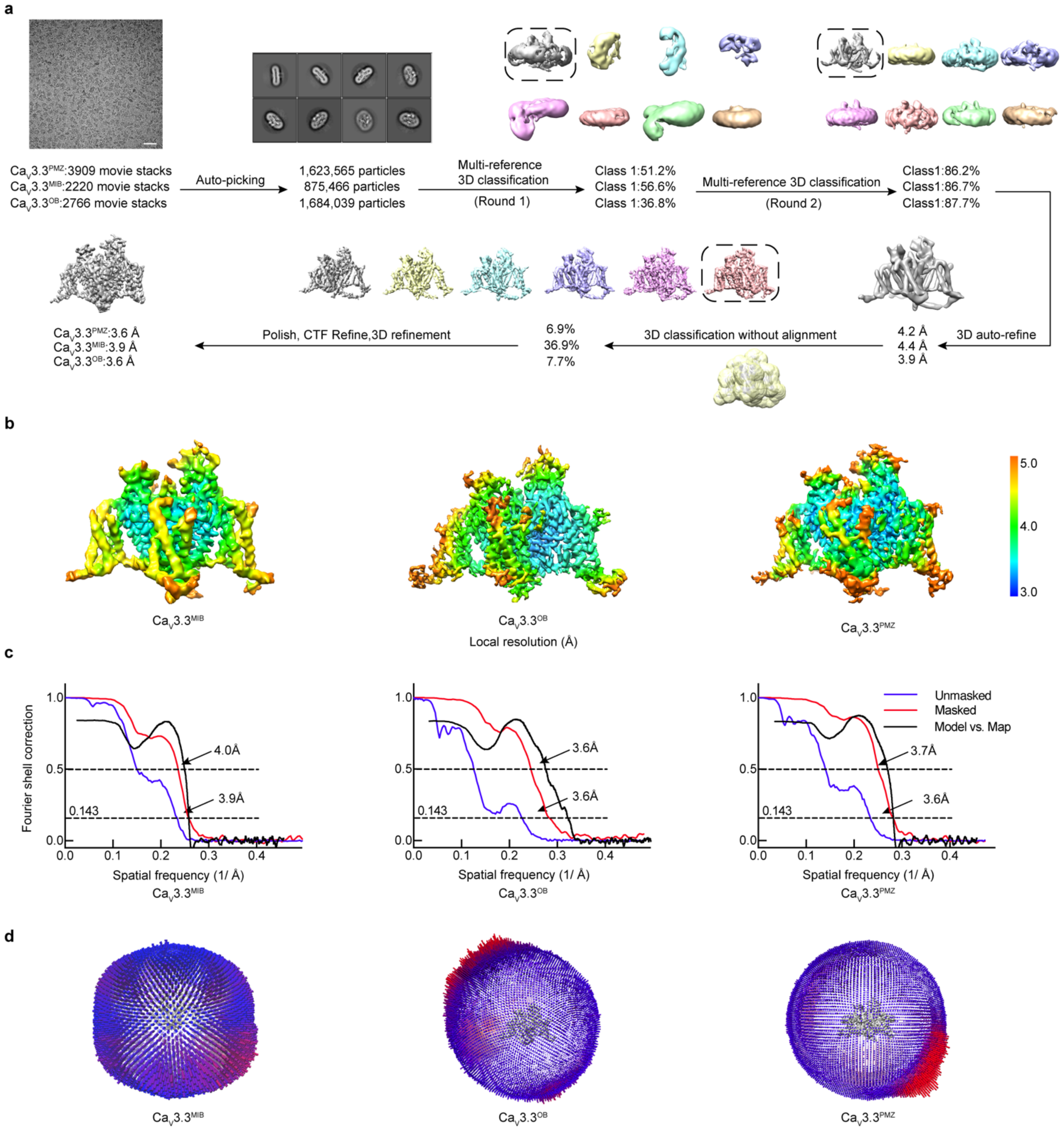

Supplementary Fig. 3 Cryo-EM data processing of $Ca_V3.3$ in complex with drugs.

a. The procedure for cryo-EM data processing. Details can be found in the image processing sections. **b.** Electron density maps colored by local resolution values. From left to right: $Ca_V3.3^{MIB}$, $Ca_V3.3^{OB}$ and $Ca_V3.3^{PMZ}$. **c.** The half-map (red) and model-map (black) Fourier shell correlation (FSC). **d.** Angular distributions of final reconstructions.

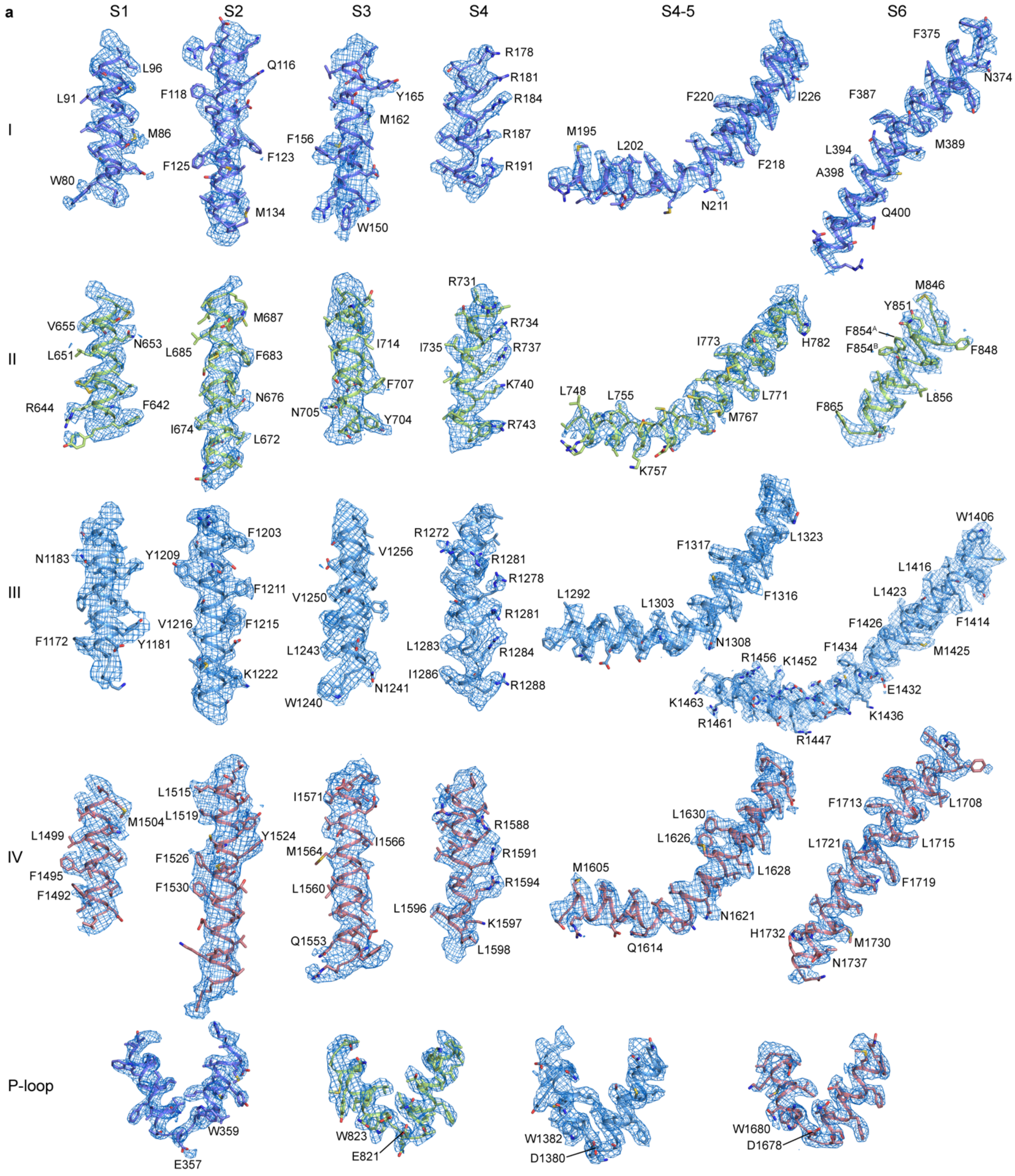

Supplementary Fig. 4 Cryo-EM map of the $Ca_V3.3^{apo}$ structure

a. The cryo-EM density map and atomic model of S1-S6 segments and pore loops in the four repeats of $Ca_V3.3^{apo}$. The side chains of key residues are labeled. The cryo-EM maps are shown as blue mesh.

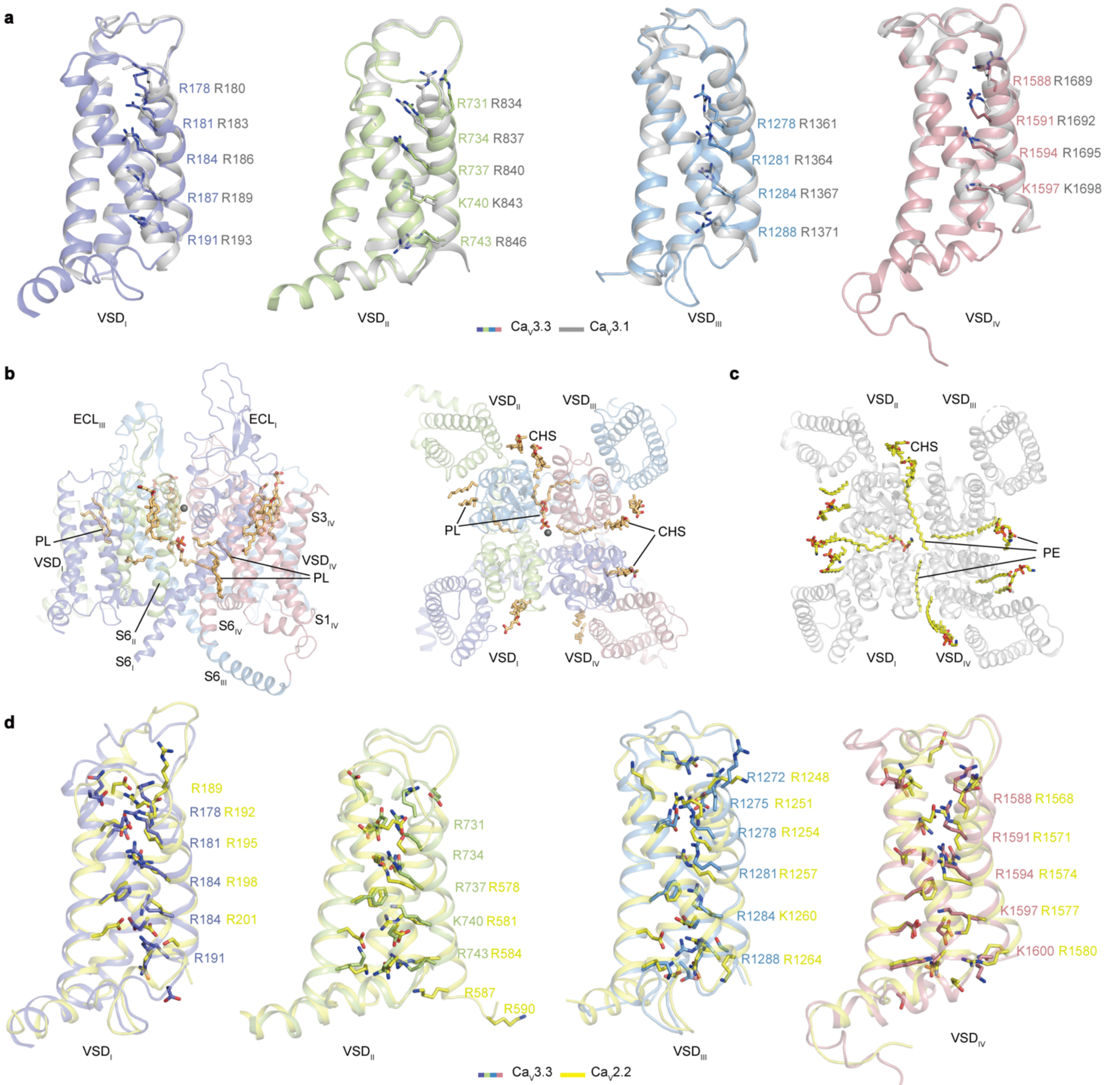

Supplementary Fig. 5 Structural comparison of the $Ca_V3.3^{apo}$ with $Ca_V3.1$ and $Ca_V2.2$ structures.

a. Structural comparison of the four VSDs in $Ca_V3.3^{apo}$ and $Ca_V3.1$ (PDB ID: 6KZO). Side views of the four VSDs of $Ca_V3.3^{apo}$ are shown in distinct colors, and $Ca_V3.1$ is shown in gray. The gating charges on S6 helix of the four VSDs are shown in sticks. **b.** Lipids bind with $Ca_V3.3^{apo}$, viewed from extracellular side and in parallel to the membrane. Phospholipids and cholesteryl hemisuccinate (CHS) are shown in sticks (orange). **c**. Lipids bind with $Ca_V3.1$ (grey), viewed from the extracellular side of $Ca_V3.1$. **d.** Interior comparison of the four VSD segments in $Ca_V3.3^{apo}$ and $Ca_V2.2$ (PDB ID: 7VFS). Side views of the four VSD channels of $Ca_V3.3$ showed in distinct colors, while $Ca_V2.2$ showed in gray. Polar residues within the

four VSDs are shown as sticks and the gating charges on S6 helices are labeled, respectively.

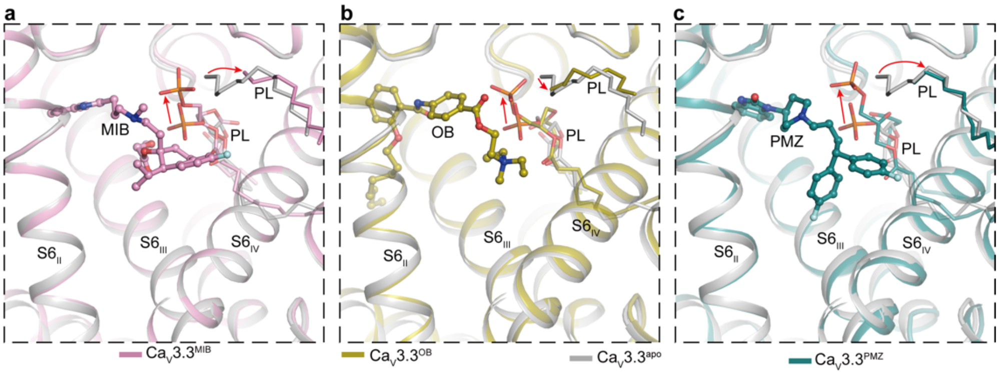

Supplementary Fig. 6 Changes in phospholipid position and upon inhibitor binding.

Structural comparison of the $Ca_V3.3^{apo}$ with structure of $Ca_V3.3^{MIB}$ (**a**), $Ca_V3.3^{OB}$ (**b**) and $Ca_V3.3^{PMZ}$ (**c**) complex, respectively. The drugs and phospholipids are shown in sticks. Displacement of the phospholipids are indicated.

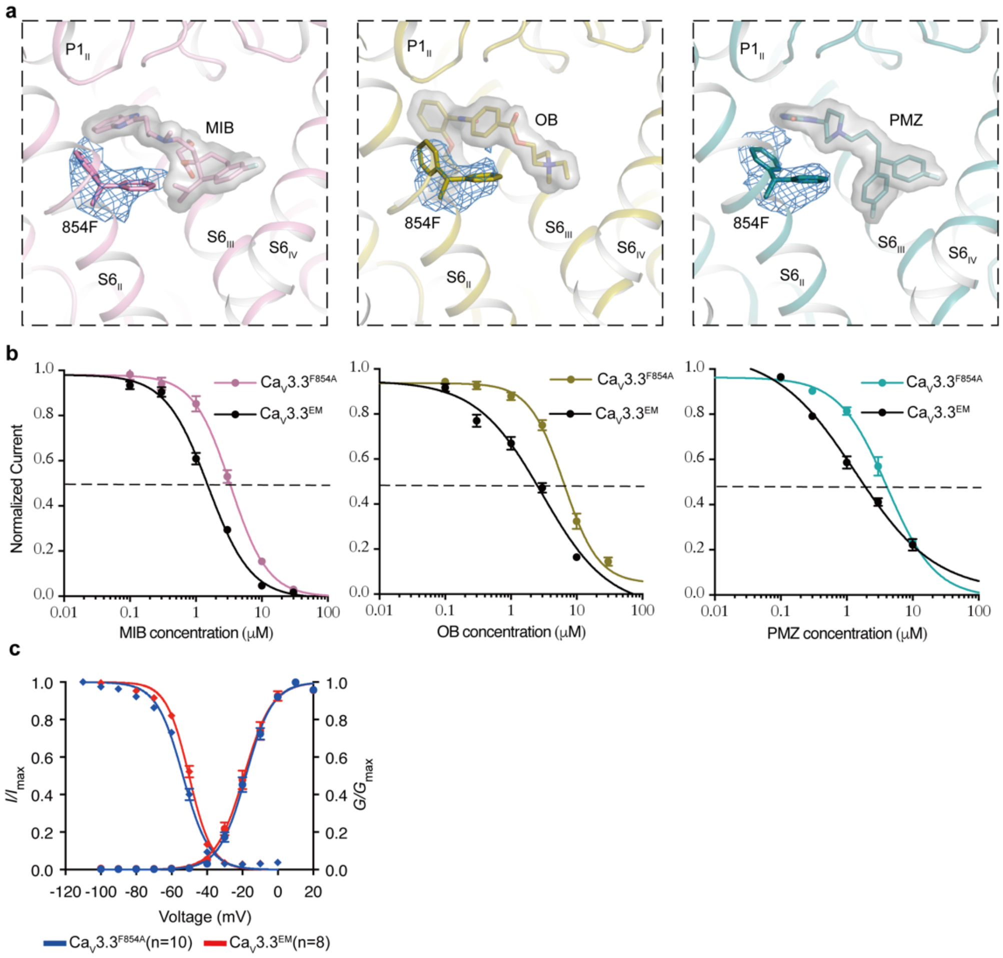

Supplementary Fig. 7 Critical interactions between F854 and drugs.

a. F854 on S6$_{II}$ shows two conformations in Ca$_V$3.3^{MIB}, Ca$_V$3.3^{OB} and Ca$_V$3.3^{PMZ}. The residues of F854 are show in sticks, overlaid with corresponding EM density colored in blue, respectively. The drugs are show in sticks and overlaid with transparent grey surfaces. **b**. The IC$_{50}$ value of MIB, OB and PMZ against Ca$_V$3.3^{F854A} exhibit higher compared to Ca$_V$3.3^{EM} (black). The sample sizes (n) tested from low to high concentration are: n=6,8,10,9,8,8 for MIB against Ca$_V$3.3^{EM}, n=5,6,8,8,8,4 for MIB against Ca$_V$3.3^{F854A}; n=6,5,8,8,5 for OB against Ca$_V$3.3^{EM}, n=5,4,8,8,7,3 for OB against Ca$_V$3.3^{F854A}; n=6,7,9,8,5 for PMZ against Ca$_V$3.3^{EM}, n=5,6,6,6,5 for PMZ against Ca$_V$3.3^{F854A}. Data are presented as mean values +/- SEM. Source data are provided as a Source Data file. **c**. Normalized conductance-voltage (G/V) and current-voltage(I/V) relationship for the Ca$_V$3.3^{EM} construct (red) and point mutation Ca$_V$3.3^{F854A} (blue). n represents the number of repeated measurements. Data are presented as mean values +/- SEM. Source data are provided as a Source Data file.

Supplementary Tables

Supplementary Table 1. Cryo-EM data collection, refinement and validation statistics

	$Ca_V3.3^{apo}$ (EMDB-32584) (PDB 7WLI)	$Ca_V3.3^{MIB}$ (EMDB-32585) (PDB 7WLJ)	$Ca_V3.3^{OB}$ (EMDB-32586) (PDB 7WLK)	$Ca_V3.3^{PMZ}$ (EMDB-32587) (PDB 7WLL)
**Data collection and processing**				
Magnification	105,000 ×	105,000 ×	105,000 ×	105,000 ×
Voltage (kV)	300	300	300	300
Electron exposure ($e^-/Å^2$)	60	60	60	60
Defocus range (μm)	−1.2 ~ −2.2	−1.2 ~ −2.2	−1.2 ~ −2.2	−1.2 ~ −2.2
Pixel size (Å)	1.04	1.04	1.04	1.04
Symmetry imposed	C1	C1	C1	C1
Initial particle images (no.)	1,140,797	875,466	1684,039	1,623,565
Final particle images (no.)	93,988	151,492	42,015	49,550
Map resolution (Å)	3.3	3.9	3.6	3.6
FSC threshold	0.143	0.143	0.143	0.143
Map resolution range (Å)	3.0 ~ 5.0	3.0 ~ 5.0	3.0 ~ 5.0	3.0 ~ 5.0
**Refinement**				
Initial model used (PDB code)	6KZO	7WLI	7WLI	7WLI
Model resolution (Å)	3.5	4.0	3.6	3.7
FSC threshold	0.5	0.5	0.5	0.5
Map sharpening *B* factor ($Å^2$)	132.8	178.3	105.6	133.1
Model composition				
Non-hydrogen atoms	9,493	9450	9461	9540
Protein residues	1,135	1135	1135	1135
Ligands	21	15	17	21
B factors ($Å^2$)				
Protein	90.31	133.68	79.53	99.39
Ligand	49.60	97.40	58.39	69.51
R.m.s. deviations				
Bond lengths (Å)	0.004	0.004	0.006	0.006
Bond angles (°)	0.690	0.929	0.778	0.911
Validation				
MolProbity score	2.08	2.14	2.05	2.05
Clashscore	12.75	16.85	12.65	12.19
Poor rotamers (%)	0.00	0.00	0.00	0.20
Ramachandran plot				
Favored (%)	92.55	92.26	93.35	92.90
Allowed (%)	7.45	6.74	6.65	7.10
Disallowed (%)	0.20	0.00	0.10	0.10

Supplementary Table 2. Primers used in this study

name	Primer sequences
$Ca_v3.3^{12Q}$-F	GAGCAGCAGCTGCAGCAACTGGAGCAGCAGCAACAGCAGGCCCAGCGG CTGCCCTACTA
$Ca_v3.3^{12Q}$-R	CCAGTTGCTGCAGCTGCTGCTCCTCTTGCTGCTGTGCCTCTTCAGCCTCC TGGTGCTGC
$Ca_v3.3^{3G}$-F	ACTTCCACAAGTGCCGGCAGCACCAGGGAGGTGGAGAGGCACGGCGGC GTGAGGAG
$Ca_v3.3^{3G}$-R	CGCCGTGCCTCTCCACCTCCCTGGTGCTGCCGGCACTTGTGGAAGTTCT CCAC
F854A-F	AACTATGTGCTCGCCAACCTGCTGGTGGCCATCCT
F854A-R	CAGGTTGGCGAGCACATAGTTGCCGAAGGTCATG

Source data of Supplementary Fig.1d

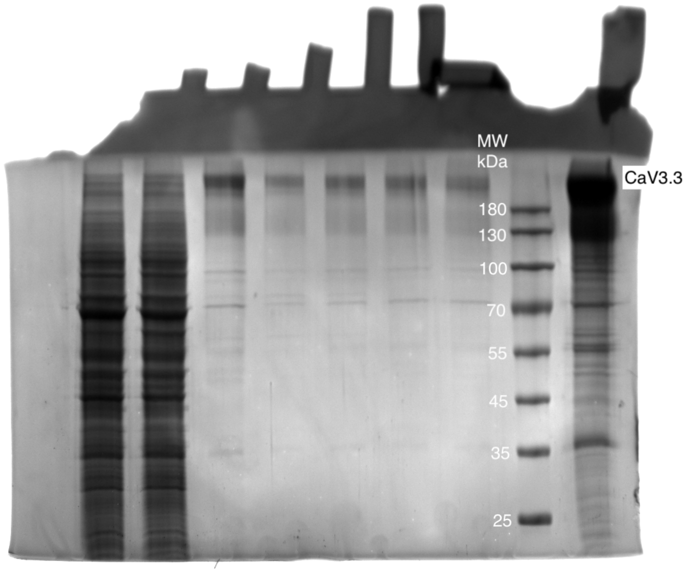

The uncropped scan of gel in Supplementary Fig.1d
