# Supplementary material for: Structure, gating, and pharmacology of human CaV3.3 channel
Source: Nat Commun. 2022 Apr 19;13:2084. doi: 10.1038/s41467-022-29728-0 (PMC9019099; doi:10.1038/s41467-022-29728-0)
Supplement: Supplementary file 3 — Reporting Summary [file 41467_2022_29728_MOESM3_ESM.pdf]

nature portfolio

Corresponding author(s): Yan Zhao
Last updated by author(s): Mar 2, 2022

Reporting Summary

Nature Portfolio wishes to improve the reproducibility of the work that we publish. This form provides structure for consistency and transparency in reporting. For further information on Nature Portfolio policies, see our Editorial Policies and the Editorial Policy Checklist.

Statistics

For all statistical analyses, confirm that the following items are present in the figure legend, table legend, main text, or Methods section.

n/a	Confirmed	
☐	☒	The exact sample size (n) for each experimental group/condition, given as a discrete number and unit of measurement
☐	☒	A statement on whether measurements were taken from distinct samples or whether the same sample was measured repeatedly
☐	☒	The statistical test(s) used AND whether they are one- or two-sided<br>*Only common tests should be described solely by name; describe more complex techniques in the Methods section.*
☒	☐	A description of all covariates tested
☒	☐	A description of any assumptions or corrections, such as tests of normality and adjustment for multiple comparisons
☐	☒	A full description of the statistical parameters including central tendency (e.g. means) or other basic estimates (e.g. regression coefficient) AND variation (e.g. standard deviation) or associated estimates of uncertainty (e.g. confidence intervals)
☐	☒	For null hypothesis testing, the test statistic (e.g. F, t, r) with confidence intervals, effect sizes, degrees of freedom and P value noted<br>*Give P values as exact values whenever suitable.*
☒	☐	For Bayesian analysis, information on the choice of priors and Markov chain Monte Carlo settings
☒	☐	For hierarchical and complex designs, identification of the appropriate level for tests and full reporting of outcomes
☒	☐	Estimates of effect sizes (e.g. Cohen's d, Pearson's r), indicating how they were calculated

Our web collection on statistics for biologists contains articles on many of the points above.

Software and code

Policy information about availability of computer code

Data collection	SerialEM 3.8, PATCHMASTER v2x90
Data analysis	Gctf 1.18, Gautomatch 0.56, cryoSPARC 3.0, RELION 3.1, PyMOL 2.5, UCSF Chimera 1.15, UCSF ChimeraX 1.11, Coot 0.9.2-pre, PHENIX 1.18.2, Origin 2019b, Excel 2016(Microsoft) GraphPad Prism 6,Adobe illustrator 2018

For manuscripts utilizing custom algorithms or software that are central to the research but not yet described in published literature, software must be made available to editors and reviewers. We strongly encourage code deposition in a community repository (e.g. GitHub). See the Nature Portfolio guidelines for submitting code & software for further information.

Data

Policy information about availability of data

All manuscripts must include a data availability statement. This statement should provide the following information, where applicable:

- Accession codes, unique identifiers, or web links for publicly available datasets
- A description of any restrictions on data availability
- For clinical datasets or third party data, please ensure that the statement adheres to our policy

human CaV3.3 cDNA sequence (UniProt ID: Q9P0X4)
map and structure of CaV3.3(PDB:7WLI;MAP:EMD-32584)
map and structure of CaV3.3 in complex with MIB(PDB:7WLJ;MAP:EMD-32585)
map and structure of CaV3.3 in complex with OB(PDB:7WLK;MAP:EMD-32586)
map and structure of CaV3.3 in complex with PMZ(PDB:7WLL;MAP:EMD-32587)
The three-dimensional cryo-EM density maps of CaV3.3apo, CaV3.3MIB, CaV3.3OB and CaV3.3PMZ have been deposited in the EM Database under the accession

codes EMD-32584[https://www.ebi.ac.uk/pdbe/entry/emdb/EMD-32584], EMD-32585[https://www.ebi.ac.uk/pdbe/entry/emdb/EMD-32585], EMD-32586[https://www.ebi.ac.uk/pdbe/entry/emdb/EMD-32586], and EMD-32587[https://www.ebi.ac.uk/pdbe/entry/emdb/EMD-32587], respectively. The corresponding coordinates for these complexes have been deposited in Protein Data Bank under accession codes 7WLI [https://doi.org/10.2210/pdb7WLI/pdb], 7WLJ [https://doi.org/10.2210/pdb7WLJ/pdb], 7WLK [https://doi.org/10.2210/pdb7WLK/pdb] and 7WLL [https://doi.org/10.2210/pdb7WLL/pdb], respectively.

Field-specific reporting

Please select the one below that is the best fit for your research. If you are not sure, read the appropriate sections before making your selection.

☒ Life sciences ☐ Behavioural & social sciences ☐ Ecological, evolutionary & environmental sciences

For a reference copy of the document with all sections, see nature.com/documents/nr-reporting-summary-flat.pdf

Life sciences study design

All studies must disclose on these points even when the disclosure is negative.

Sample size	Sample sizes were not predetermined for this study. The amount of micrographs is determined by the microscope time. For the electrophysiology experiment, no statistical methods were used to predetermine sample sizes but our sample sizes are similar to those reported previously in the field. See Methods, 'Whole-cell Voltage-clamp recordings' subsection.
Data exclusions	No data was excluded from analysis.
Replication	Sample preparation related experiments including purification, SDS-PAGE gels were reproduced at least three times independently. Electrophysiological experiments were repeated a minimum of 3 times across multiple days and transfection batches. All attempts at replication produced similar results.
Randomization	Sample purification were repeated several times with different batches of cells. Square of grids for cryo-EM data collection were randomly selected. Cells for electrophysiology experiment with GFP fluorescence was randomly selected.
Blinding	The investigators were not blinded as the parameters for cryo-EM analysis and electrophysiology experiments did not require subjective assessments of the treatments or their outcomes that might otherwise influence the validity of the results.

Reporting for specific materials, systems and methods

We require information from authors about some types of materials, experimental systems and methods used in many studies. Here, indicate whether each material, system or method listed is relevant to your study. If you are not sure if a list item applies to your research, read the appropriate section before selecting a response.

Materials & experimental systems

n/a	Involved in the study	
☒	☐	Antibodies
☐	☒	Eukaryotic cell lines
☒	☐	Palaeontology and archaeology
☒	☐	Animals and other organisms
☒	☐	Human research participants
☒	☐	Clinical data
☒	☐	Dual use research of concern

Methods

n/a	Involved in the study	
☒	☐	ChIP-seq
☒	☐	Flow cytometry
☒	☐	MRI-based neuroimaging

Eukaryotic cell lines

Policy information about cell lines

Cell line source(s)	HEK293 cell line(Gibco, USA),FreeStyle 293-F cells (Gibco, USA); 293-T cells (Gibco, USA)
Authentication	Not authenticated.
Mycoplasma contamination	The cells were tested negative for mycoplasma contamination.
Commonly misidentified lines (See ICLAC register)	Not commonly misidentified lines were used.
